# A laboratory pilot study on voids in flowable bulk-fill composite restorations in bovine Class-II and endodontic access cavities after sonic vibration

**DOI:** 10.1038/s41598-023-45836-3

**Published:** 2023-10-29

**Authors:** Philipp Körner, Sandra C. Gerber, Cindy Gantner, Blend Hamza, Florian J. Wegehaupt, Thomas Attin, Shengjile Deari

**Affiliations:** 1 Clinic of Conservative and Preventive Dentistry, Center of Dental Medicine, University of Zurich, Plattenstrasse 11, CH-8032 Zurich, Switzerland; 2 Clinic of Orthodontics and Pediatric Dentistry, Center of Dental Medicine, University of Zurich, Plattenstrasse 11, CH-8032 Zurich, Switzerland

**Keywords:** Dental materials, Dental treatments, Restorative dentistry

## Abstract

This pilot study investigated whether sonic-powered application of a bulk-fill resin-based composite (RBC) in Class-II or endodontic access cavities reduces void formation. The crowns and roots of 60 bovine teeth with Class-II cavities (C) and endodontic access cavities (E) respectively, were assigned to ten groups (C1–C5, E1–E5). Cavities were filled with RBC (SDR flow + , one increment) using different application techniques: no adaptation (C1 + E1), spreading of RBC on the cavity surfaces with a dental explorer tip (C2 + E2), low (C3 + E3) or high frequency (C4 + E4) direct activation by inserting a sonic-powered tip into RBC and high frequency indirect activation with an ultrasonic insertion tip (C5 + E5). The restorations were light-cured and investigated for voids using microtomography. The number of voids and percentage of voids related to the volume were statistically analysed (*α* < 0.05). While most voids in Class-II restorations were observed in C4 (*p* ≤ 0.0031), no significant differences were found between the other groups (*p* > 0.05). The percentage of voids showed no differences in E1-E5 (*p* > 0.05). C4 showed a significantly higher percentage of voids compared to C2 (*p* < 0.001). There is no benefit in applying sonic vibration when filling Class-II or endodontic access cavities.

## Introduction

Resin-based composites (RBC) have become the material of first choice for direct tooth restoration in modern dentistry, covering a wide range of indications and restorative treatment options. They are commonly used for all types of tooth restorations in both the anterior and posterior region^1^. For example, tooth-colored RBC can be used to seal and fill endodontic access cavities (EAC) after root canal treatment, allowing for esthetic rehabilitation and reducing the need of dental crowns, particularly in anterior teeth^2,3^.

**Figure 1:**
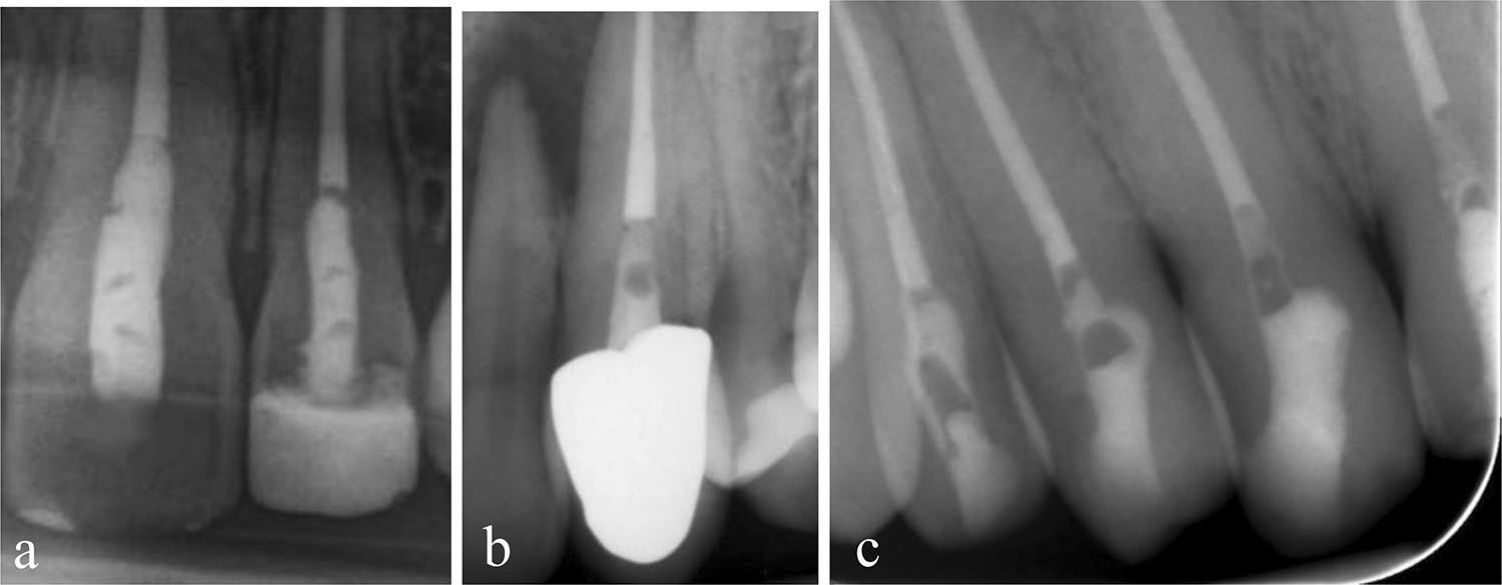
(**a**) Insufficient adaptation between the increments inside the restorations of two EAC cavities after using packable RBC. (**b**) Void inside the filling of an EAC after using a flowable bulk-fill RBC. (**c**) Entrapped air on the cavity floors and inside the restorations of multiple EAC.

Conventional firm, packable, high viscosity RBC, as well as low viscosity, flowable RBC are frequently used in this context. However, they require multiple increments of ≤ 2 mm thickness, to ensure adequate polymerization, minimize polymerization shrinkage and stress. Proper application requires more time and for packable RBC, there is a risk of insufficient adaptation to the cavity surfaces and also between the increments (Fig. 1a)^4^.

To avoid these potential shortcomings, flowable bulk-fill RBC has been developed and has gained great popularity among dentists^5^. It can easily be applied and adequately polymerized in increments of up to 3–5 mm layer thickness, while maintaining low polymerization shrinkage and stress^6^. These properties are enabled by novel resins, specific fillers, polymerization modulators and optimized photo-initiator systems^7^. Superior flow and adaptation properties are claimed by some manufacturers. However, an increased number of voids in the cured material (Fig. 1b) have been observed and reported in clinical practice^8–10^, especially after the use of flowable RBC. The reason for this may be attributed to pre-existing air inclusions within the RBC before application or by an insufficient application technique leading to an inhomogeneous distribution of the material^11^. It is also possible that applied low viscous RBC may cover and trap air on the cavity floor, resulting in the formation of voids (Fig. 1c). Despite esthetic shortcomings in the X-ray control, minor internal voids are unlikely to affect the clinical outcome and durability of the restoration^12^. However, with increasing size, quantity or unfavorable location of the voids, combined with frequent pressure changes within the air inclusion, the mechanical stress on the filling material might also increase, thus enhancing the susceptibility to fracture, micro-leakage and discoloration^13–15^.

To implement controlled application and void reduction, some clinicians recommend adapting and spreading flowable RBC onto the cavity surface using the tip of a dental explorer^16,17^. This way, energy is imparted to the surface of the flowable RBC, which reduces surface tension and improves the wettability and adaptation to the cavity surface. However, experiences in clinical practice do not show reliable and evident prevention of voids using this technique^18^.

Vibrating sonic tips are routinely used in endodontics nowadays to stimulate the rinsing solution within the root canal and enable better cleaning of the canal system^19,20^. The tips vibrate at high frequency, creating an upward flow within the rinsing liquid, while washing tissue residue and entrapped air to the surface. Keeping this in mind, this technique might be transferred to the application of flowable RBC, in order to loosen potential air inclusions and mobilize them to the surface. Moreover, sonic vibration might impart energy into the applied and uncured flowable RBC, thus modifying its rheological properties to decreased viscosity, which might result in better adaptation of the material to the cavity surface^21^.

Sonic vibration might be able to be applied in a direct manner by using an endodontic plastic tip that is dipped into the uncured RBC before activation, similar to the endodontic stimulation of rinsing solution. Alternatively, applying sonic vibration to the entire tooth, including the uncured RBC, may indirectly mobilize air, similar to the mode of action of a lab plaster vibrator.

Taking this into consideration, the aim of this pilot study was to investigate whether direct or indirect sonic-powered application of flowable bulk-fill RBC in Class-II cavities or EACs in vitro can reduce voids. The null hypothesis was that sonic vibration does not affect voids in flowable bulk-fill RBC restorations of Class-II cavities or EACs.

## Methods

### Sample preparation

A total of 60 freshly extracted and cleaned permanent lower incisors from 2–3-year-old cattle were separated at the cemento-enamel junction using a low-speed saw (IsoMet; Buehler, Lake Bluff, IL, USA). Standardized Class-II cavities were prepared in the crowns and the root canals were shaped, obturated with gutta-percha and prepared with standardized access cavities as follows:

### Class-II cavities

**Figure 2:**
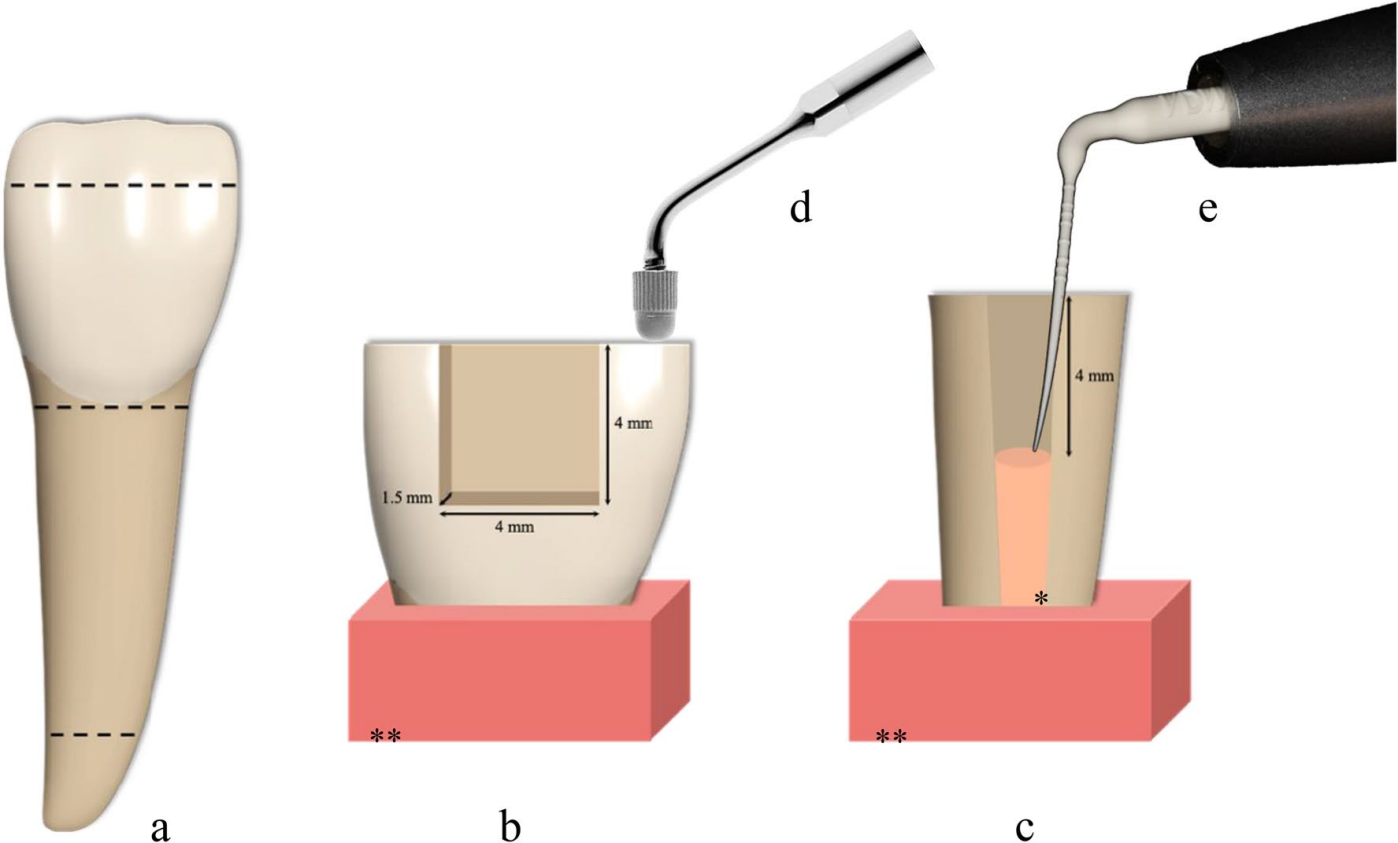
(**a**) Sectioning of the bovine front teeth, cavity design and dimension of the prepared and embedded Class-II cavities (**b**) and EAC (**c**). (**d**) Example for indirect activation of the applied RBC by touching the tooth close to the cavity margin with an ultrasonic insertion tip. (**e**) Example for direct activation by inserting a sonic-powered tip into the applied RBC. *Gutta-percha, **Acryl resin.

An illustration of sample preparation and cavity design is given in Fig. 2. The incisal edge of the crowns was reduced by a quarter of the height using the IsoMet low-speed saw. The surfaces were then smoothed using a water-cooled carborundum disc (Tegramin 30 SiC foil #220; Struers, Birmensdorf, Switzerland) and the bovine-specific cementum layer on the enamel surface was removed. The crowns were aligned horizontally at the incisal edge and vertically on the approximal surface and embedded in acryl resin (Paladur; Kulzer, Hanau, Germany). Next, standardized Class-II cavities with a height of 4 mm, a width of 4 mm and a depth of 1.5 mm were prepared using a water-cooled diamond bur (Garant D126; Hoffmann, Munich, Germany) mounted on a fixed milling device (BFW 40/E; Proxxon, Foehren, Germany) and the edges were bevelled by 0.5 mm (SonicFlex tips No. 58/59; KaVo, Biberach/Riss, Germany). Each prepared specimen was then sprayed with water for 5 s and fitted with a steel matrix band (Hawe 499 B; Kerr, Bioggio, Switzerland) which was secured with an elastic band (Manufix sensitive XS (cut); B. Braun, Melsungen, Germany) and duct tape (Scotch transparent; 3 M, Saint Paul, MN, USA) to ensure a gap-free adaptation.

### Endodontic access cavities

An illustration of the sample preparation and cavity design is shown in Fig. 2. The roots were shortened at the coronal end to a length of 10 mm using a low-speed saw. The root canals were then instrumented with rotary endodontic files (ProFile ISO 40; Dentsply Maillefer, Ballaigues, Switzerland), rinsed with water, dried with paper tips (ProTaper Next X4; Dentsply Sirona, Charlotte, NC, USA) and obturated with gutta-percha (Conform Fit for ProTaper Next X4; Dentsply Sirona and PN 822–602; Obtura Spartan, Algonquin, IL, USA) using a thermoplastic obturation system (SuperEndo Alpha II and Beta; B&L Biotech, Fairfax, VA, USA). To imitate the basal area of the cavity in anterior teeth and premolars, standardized cavities of 4 mm depth were prepared using a tapered drill that was shortened at the tip to a length of 4 mm (D.T. Light-Post Drill #3; VDW, Munich, Germany). The prepared specimens were rinsed with water for 5 s and also embedded in acryl resin (Paladur).

Finally, the 60 tooth roots with shaped and obturated root canals and prepared EACs (E) and the 60 crowns with prepared and matrix-fitted Class-II cavities (C) were randomly assigned to ten groups (E1–5 and C1–5, n = 12 per group). All specimens were stored at 5 °C in 0.1% thymol (1.08167.1000; Merck, Darmstadt, Germany). The experimental protocols and methods were conducted in accordance with ARRIVE guidelines^22^. The bovine teeth are classified as slaughter by-products and are therefore free from ethical and animal welfare concerns.

### Application of the flowable bulk-fill resin-based composite

All specimens were sprayed with water for 5 s and dried with paper tips (E) or gently air dried (C). Afterwards, a universal adhesive (Scotchbond Universal Adhesive; 3 M, Seefeld, Germany) was applied with a microbrush (applicator tip 50,456–1; Dentsply DeTrey, Konstanz, Germany) for 20 s in self-etch mode. Excess adhesive was removed with a paper tip and the solvent was gently air dried for 5 s before the adhesive was light-cured for 10 s using a polywave LED curing unit (Bluephase G2; Ivoclar, Schaan, Liechtenstein) with a 385–515 nm emission spectrum and an output irradiance of at least 1.1 mW/cm^2^, as verified using a radiometer (Cure Rite Efos model #8000; Efos, Mississauga, Canada). The cavities were then filled with a bulk-fill RBC (SDR flow + ; Dentsply Sirona) in one increment, with the cannula of the compule slowly withdrawn from the bottom of the cavity while consistently keeping the tip within the applicated material. For each sample, a new compule of RBC was used without disposing the first amount of RBC before dispensing it into the cavity. In the control groups (C1 and E1), the applied material was not manipulated before light-curing. In C2 and E2, the RBC was manually adapted to the cavity surfaces using a sharp dental explorer tip (Double-ended explorer EXD5 Gr #31 round; Hu-Friedy, Chicago, IL, USA).

In C3 and E3 (low frequency) and C4 and E4 (high frequency), a sonic-powered tip (EDDY sonic-powered endo irrigation; VDW) was shortened to 5 mm, the thickness of a conventional dental explorer (0.6 mm), and inserted into the uncured RBC material that was initially filled into the cavity, as described for C1 and E1. Subsequently, the tip was activated for 5 s at a sonic intensity of 160,000 units (C3 and E3) or 230,000 units (C4 and C5), as displayed on the treatment unit (Estetica E70 Vision; KaVo, Laufen, Germany). The small amount of RBC remaining on the tip of the compule after deactivation was disposed of and refilled without additional activation before light-curing.

**Figure 3:**
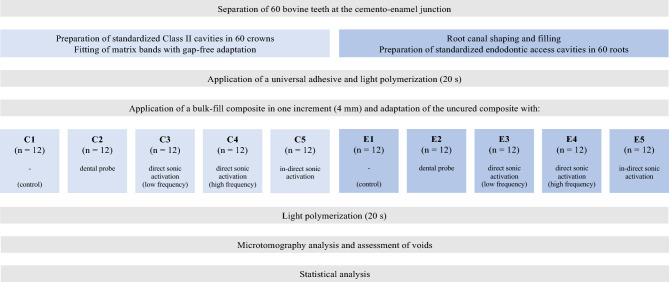
Experimental design.

In C5 and E5, sonic vibrations were indirectly transmitted to the RBC by placing an ultrasonic insertion tip (Piezo Cem Tip 225; KaVo) on the tooth surface next to the cavity margin (Fig. 2d), followed by the activation for 5 s at the highest sonic level. Immediately after the respective treatment procedure all samples were light-polymerized for 20 s using a polywave LED curing unit (Bluephase G2) at an output irradiance of at least 1.1 mW/cm^2^. The distance between the curing unit tip and the uncured RBC was approximately 1 mm. The filling procedures of C and E were each performed by one investigator. The study design is illustrated in Fig. 3.

### Microtomography (µCT) analysis and assessment of voids

**Figure 4:**
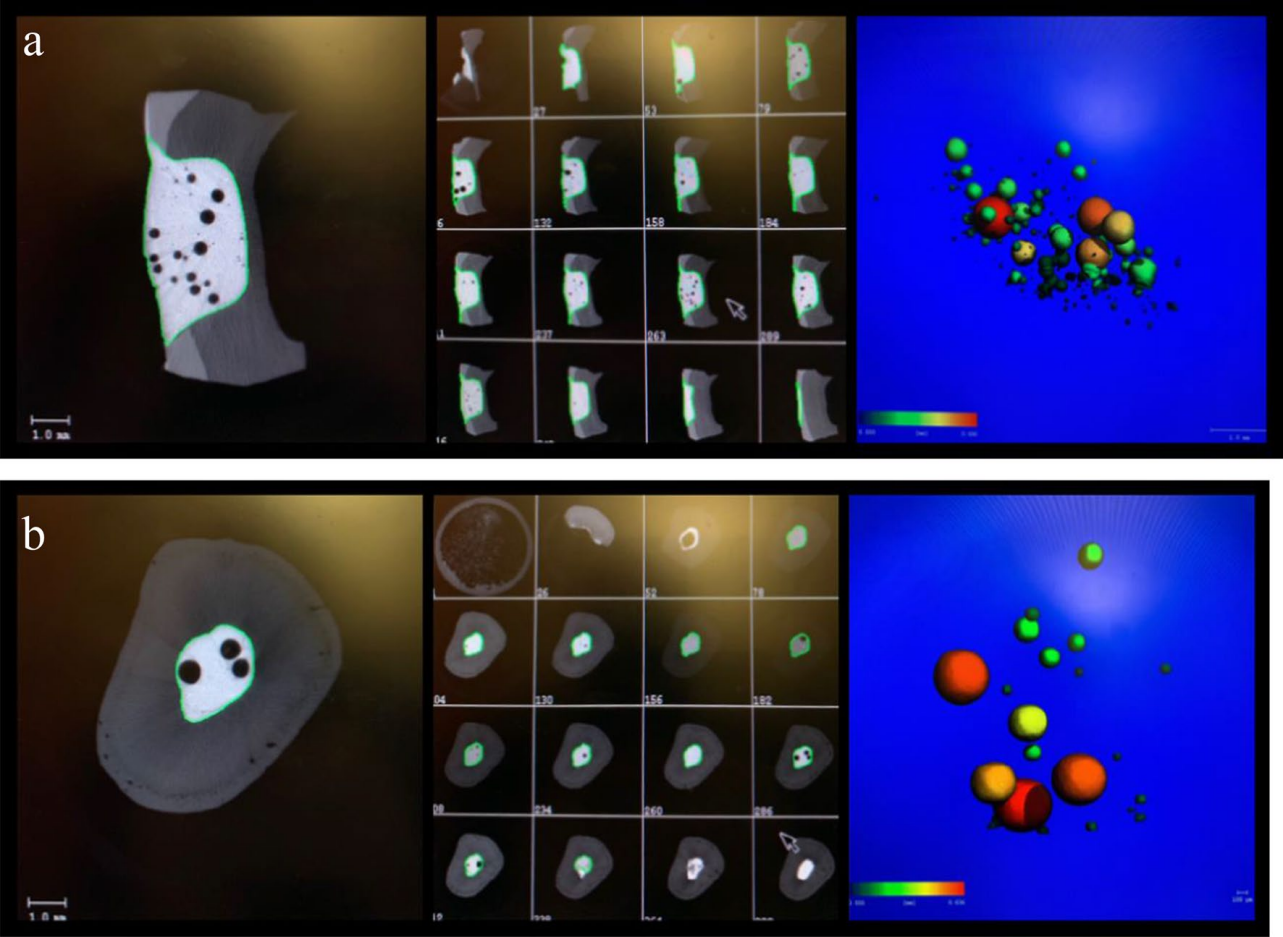
µCT analysis of a Class-II (**a**) and an EAC filling (**b**) with a flowable bulk-fill RBC. The middle and left images show virtual vertical slices of the filling with multiple air inclusions. The filling margin is marked green. A three-dimensional reconstruction of voids in the filling (color-coded by size) is illustrated in the right images.

For each group, a blinded and calibrated investigator assessed the number, location and percentage of voids related to the overall volume of the filling using µCT (lCT40; Scanco Medical, Basserdorf, Switzerland). The location of voids was categorized as internal (within the filling), marginal (at the interface of the filling and the tooth) and superficial (on the surface of the filling). In accordance with a previously established method^23,24^, the samples with filled EAC and Class-II cavities were placed in a sample holder and scanned at 70 kV and 114 µA with an isotropic resolution of 12 µm, resulting in 800 to 1200 slices per sample. Virtual models of the RBC restorations were generated using specialized software (IPL V6.6B; Scanco Medical) based on slice-by-slice reconstruction of the µCT scans, allowing for quantitative assessment of the total volume and the void volume of each filling. The scan resolution predefined the minimal ascertainable inhomogeneity (air inclusion) within a filling. While all measurable inhomogeneities were included in the assessment of the percentage of voids related to the overall volume of the filling, only air inclusions ≥ 50 µm were counted for the assessment of the number and the location of the voids. This was due to the exceedingly high and innumerable number of miniscule voids observed in some restorations. An example for the µCT analysis of a Class-II filling and an EAC filling, including a three-dimensional reconstruction of voids, is presented in Fig. 4.

### Statistical analysis

Descriptive statistics were computed for the target variables (number, location and percentage of voids related to the overall volume of the filling). Additionally, the target variables “number of voids “ and “percentage of voids “ were investigated using statistical inference. The non-parametric omnibus test Kruskal–Wallis one-way analysis of variance was used to assess if the test groups originated in a common population. The non-parametric Conover test was carried out as a post-hoc test to check for significant differences between the test groups and the target variables. To account for multiple testing, corrections were made according to the Bonferroni-Holm method. The level of significance was set at 5%. One blinded statistician performed the entire statistical analysis and plots using the statistical software R (2015) (R: A language and environment for statistical computing; R Foundation for Statistical Computing, Vienna, Austria)^25^, including the packages tidyverse^26^, rcompagnion^27^, ggplot2^28^ and PMCMR^29^. A power analysis was not possible since no comparable data were available prior to the study. However, in a similar study conducted at the institute^30^, a total of 60 cavities were prepared and allocated to 10 experimental groups. Based on that study, it was decided to use a slightly higher number, to ensure a sufficient number of samples. Nevertheless, this study must still be regarded as a pilot study.

## Results

### Number of voids

Overall, air entrapment was detected in 93.3% of the Class-II and in 95.0% of the EAC restorations.

**Figure 5:**
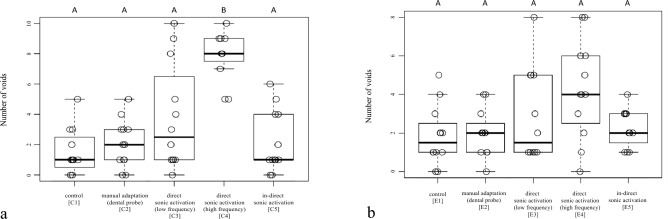
Boxplot with number of voids per sample in the restorations of the (**a**) Class-II cavities (C1–5) and (**b**) EAC (E1–5). The horizontal line in the box represents the median value, the box represents the 25th and 75th percentile and the whiskers represent the 5th and 95th percentile. Not significantly different values are marked with same capital letters.

Within the restorations of the Class-II cavities, the following numbers of voids [median (interquartile range)] were observed: C1 [1.0 (1.5)], C2 [2.0 (2.0)], C3 [2.5 (4.8)], C4 [8.0 (1.2)], C5 [1.0 (3.0)] (Fig. 5a). While no significant differences were found between the test groups C1, C2, C3 and C5 (*p* > 0.05), the number of voids was significantly higher after high frequency direct activation (C4) (*p* ≤ 0.0031). Within the EAC restorations, no significant differences (*p* > 0.05) were found between the test groups (E1–E5): E1 [1.5 (1.2)], E2 [2.0 (1.2)], E3 [1.5 (4.0)], E4 [4.0 (3.2)], E5 [2.0 (1.2)] (Fig. 5b).

### Percentage of voids related to the overall volume of a filling

**Figure 6:**
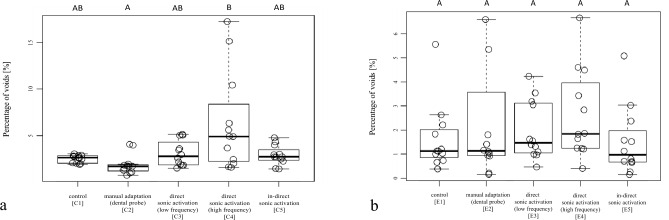
Boxplot with the volume percentage of voids [%] per sample in the restorations of the (**a**) Class-II cavities (C1–5) and (**b**) EAC (E1–5). The horizontal line in the box represents the median value, the box represents the 25th and 75th percentile and the whiskers represent the 5th and 95th percentile. Not significantly different values are marked with same capital letters.

In the restorations of the Class-II cavities, the following percentages of voids (%) [median (interquartile range)] were recorded: C1 [2.7 (0.8)], C2 [1.7 (0.6)], C3 [2.8 (2.1)], C4 [4.9 (5.0)], C5 [2.7 (0.8)]. No significant differences were found between the test groups C1, C2, C3 and C5 and between C1, C3, C4 and C5. The test group C2 showed a significantly lower percentage of voids compared to C4 (*p* < 0.001) (Fig. 6a).

Regarding the restorations of the EAC, no significant differences in percentage of voids (%) [median (interquartile range)] were found between the five test groups E1 [1.1 (1.0)], E2 [1.1 (1.8)], E3 [1.5 (2.0)], E4 [1.8 (2.5)], E5 [1.0 (1.1)] (*p* > 0.05) (Fig. 6b).

### Location of voids

An overview on the location of voids is given in Table 1. All groups presented internal and marginal voids and a comparatively low number of voids located on the surface of the restorations. The most voids were found inside the restorations after high frequency direct sonic application in the Class-II cavities (C4).

**Table 1:** Location of voids (total per group) in the restorations of Class-II cavities and EAC for the different application techniques.

Group	Location of voids (total per group)		
	Internal	Marginal	Superficial
Class II cavities			
C1 No adaptation (control)	8	8	2
C2 Manual adaptation with dental explorer	16	6	3
C3 Direct sonic activation (low frequency)	35	9	1
C4 Direct sonic activation (high frequency)	80	18	0
C5 Indirect sonic activation	8	17	1
Endodontic access cavities			
E1 No adaptation (control)	19	3	0
E2 Manual adaptation with dental explorer	18	5	1
E3 Direct sonic activation (low frequency)	14	18	2
E4 Direct sonic activation (high frequency)	21	30	0
E5 Indirect sonic activation	23	4	0

## Discussion

Partly rejecting the null hypothesis, the results showed that sonic vibration had a significant effect on voids in flowable bulk-fill RBC restorations of Class-II cavities, with significantly higher numbers and percentages of voids observed when high frequency, direct sonic vibration was applied. In contrast, no significant differences in the number or percentage of voids related to the volume of the restorations of EACs were found between the different application techniques^31^.

Prior to the study, no power analysis was performed as no comparable data were available. In a study with a similar design^30^ a total of 60 cavities were prepared and allocated to 6 experimental groups. Based on this, it was decided to use two additional samples in each group. However, when looking at the results of the present study, it becomes apparent that the sample size may still have been insufficient. For example, Fig. 5b shows that groups E1, E2 and E5 do not show significant differences compared to E4, despite the fact that the average number voids in E4 is more than twice as high as in the other groups. It is therefore reasonable to assume that the study was underpowered, which must be recognized as a limitation. However, this limitation is the result of the nature of a pilot study, which is characterized by the lack of pre-existing data, making it impractical to conduct a power analysis. Consequently, the data obtained can be used as an input for future studies to calculate sample sizes.

The Class-II cavities and EACs in this study were prepared in extracted lower bovine incisors. Bovine teeth are easily accessible, have been previously used and discussed in multiple studies and can therefore be regarded as suitable substitutes for human teeth, within the scope of this study^11,32^. The cavity dimensions aimed to reflect realistic conditions which may occur during root canal treatments of anterior teeth and premolars, as well as Class-II cavity restorations. It is worth noting that while the definition of a Class-II cavity originally refers to posterior teeth according to Black^33^, the cavities in the present study were prepared in modified anterior bovine teeth, due to the inadequate shape and anatomy of posterior bovine teeth, which are generally not suitable for standardized Class-II cavities.

All cavities were treated with a universal adhesive applied in the self-etch mode i.e. without additional phosphoric acid etching. Aiming to minimize potential errors and facilitate the application, this technique was found to be the most suitable, within the scope of this study, as it has been shown that there are hardly any differences compared to current multi-step etch-and-rinse adhesives^34^. A new compule of RBC was used for each sample to avoid changing the compule while filling up the cavity, thus minimizing the risk of incorporating additional air between non-polymerized increments. However, in groups where a dental explorer or ultrasonic tips were inserted into the applied RBC (C2–4, E2–4), the cavities were carefully refilled if RBC was removed from the cavity while withdrawing the instruments. Therefore, it is not feasible to exclude the possibility of additional air being entrapped in the filling, which might be a limitation of the study. Furthermore, it should be noted that the experiments were performed by two master students, with limited clinical experience, but under the guidance and supervision of experienced clinicians.

The three-dimensional evaluation of the samples using µCT is generally regarded as a suitable tool for non-destructive porosity assessment of RBC restorations. This method not only provides high-resolution images and a detailed assessment of the number and size of voids, but also allows for precise determination of the void volumes, including the percentage related to the volume of the respective filling^21^. This can be considered a strength of this study. The µCT analysis and assessment of voids was based on established and proven methodical approaches conducted in previous studies at the institute and was performed by one blinded investigator who was instructed and calibrated by an experienced colleague with superior expertise in µCT analysis. As this process was implemented during data analysis and no further calibration was performed using independent samples, there is still an inherent risk of calibration bias, although this would have affected all test groups equally as the investigator was blinded.

The aim of this study was to evaluate the potential of sonic-powered application of a flowable bulk-fill RBC in the restorations of Class-II cavities or EACs, to reduce voids. In theory, the vibrations would create an upward flow within the uncured RBC, thus loosening air inclusions and vibrating them to the surface. Additionally, the enhanced thixotropy of the RBC during activation would promote better adaptation to the surfaces^31^.

Overall, voids were detected in 93.3% (Class-II) and 95.0% (EAC) of all restorations, which is surprisingly high. Even in the best performing group (E1), 83.3% of the restorations were affected. Another study that investigated voids in Class-II cavities using optical coherence tomography reported similar high percentages of voids (79.2%) in restorations filled with a flowable bulk-fill RBC^10^. Inevitably, questions arise about the reason for these high numbers. It should be taken into account that due to the particularly high resolution (12 µm) of the µCT, even the tiniest air inclusion, which normally cannot be seen on an X-ray or without magnification, could be assessed in this study, which might explain these high numbers. While all measurable inhomogeneities were counted in the assessment of the percentage of voids related to the overall volume of the filling, only air inclusions ≥ 50 µm were counted for the assessment of the number and location of the voids in this study. While this may be considered a limitation of the study, this decision was made due to the occasionally innumerable amounts of miniscule voids or inhomogeneities in various restorations, as illustrated in Fig. 4a.

An attempt was made to identify the sources or causes of the inhomogeneities. It is conceivable that air inclusions may be present in the RBC compules prior to their use, due to the manufacturing processes. These air inclusions could then in turn transfer to the filling as soon as the RBC is applied^35^. Additionally, air may be aspirated through the cannula of the compule-tip during inconsistent or intermittent composite application. In such cases, it might be helpful to squeeze out and dispose of a small amount of RBC before dispensing it into the cavity. However, as a new compule of RBC was used for each filling and no benefits of this technique have been described in literature, the RBC was applied into the cavity without disposing of the first drop.

Previous studies confirm that the way a flowable RBC is applied into the cavity has an influence on the formation of voids^12,36^. In particular, in small, deep and tapered cavities, inhomogeneous distribution of low viscous RBC flowing down the lateral cavity surface might trap and enclose air at the bottom of or within the cavity, thus forming voids^37^. As confirmed in this study, a considerable number of voids are present in restorations without any further manipulation of the RBC after application. It was therefore of interest to investigate if these voids could be eliminated through the use of additional adaptation techniques including the manual spreading of the applied RBC to the cavity surfaces with a sharp dental explorer, the use of low or high frequency direct activation by putting a sonic-powered tip into the applied RBC, or the use of high frequency indirect activation by setting the whole tooth under vibration using an ultrasonic insertion tip.

It was found that none of the investigated additional adaptation techniques provided a benefit in terms of void-reduction. On the contrary, high frequency, direct sonic-powered application resulted in significantly higher numbers of voids in Class-II cavities. Overall, there was a clear tendency towards increased void formation with increased frequency of direct sonic activation for the restorations of Class-II cavities and EACs. Similarly, other studies indicate that sonic-activated application might lead to increased air inclusions or void formation in RBC restorations^8,21^. It is conceivable that the vibrating tips might displace the honey-like flowable RBC for a fraction of a second, leading to a sort of air crater along the tip that rapidly collapses on the surface, thus incorporating air within the RBC and forming new air inclusions. With increased frequency, this “foaming effect” seems to intensify, which might explain the results observed. When comparing the Class-II cavities with the EACs, differences can mainly be found in the high frequency, direct sonic-powered application groups. It might be speculated that the reason for the higher numbers and percentage of voids in the Class-II cavities could also be due to the above described “foaming effect” taking place in different cavity geometries. As the Class-II cavities had a larger width of 4 mm (compared to the EACs with 1.8–2.1 mm diameter), this could have allowed for a greater vibration amplitude of the tip without contact to the lateral cavity walls. Thus, more RBC might have been displaced, resulting in more extended collapsing air craters and hence more air inclusions.

Analogous to a laboratory plaster vibrator, the indirect activation aimed to loosen air inclusions and vibrate them to the surface by placing an ultrasonic insertion tip on the tooth surface next to the cavity margin. As the results in this study did not show any benefit for this technique, it might be speculated that the rheological properties of the RBC did not allow the voids to move upwards to the surface, or that the sonication process may have caused smaller air inclusions (already present in the material) to coalesce and form consolidated, more obvious voids^21^.

Compared to the control group, manual adaptation of the RBC to the cavity surfaces also did not result in a reduced number of voids in both cavity geometries, nor did it reduce the percentage of voids related to the volume in the restorations of the EACs. However, the overall lowest percentage of voids was found in Class-II cavities with manual adaptation. On the one hand, this technique might help to adapt the RBC to the cavity walls and remove obvious air inclusions within and at the surface of the material, which can be imagined to be more efficient for larger and easily accessible cavity geometries. On the other hand it seems questionable whether voids, especially those located at the bottom of small, deep and visually uncontrollable cavities, can be reliably eliminated or moved to the surface and then eliminated with a sharp dental explorer. Moreover, there is an additional risk of entrapping air in the RBC, thus generating new or even worse air inclusions^38^.

The manufacturer of the flowable bulk-fill RBC used in this study claims that their product has superior flow and self-leveling properties and does not require any additional instrumentation or manipulation. This claim was supported by the low numbers and percentages of air inclusions in the control group. Nevertheless, voids were detected in 83.3% of all restorations, which may be attributed to either air inclusions already present in the RBC compule or the way the RBC was applied into the cavity, especially with regard to air getting enclosed and trapped. To further minimize the risk of air inclusions, it is recommendable to fill up the cavity starting at the bottom and slowly lifting the tip of the compule simultaneously with the rising surface level, especially in small, deep or visually uncontrollable cavities^39,40^.

## Conclusion

For the present pilot study, the following conclusions can be made:

- There is no reduction of voids by applying direct or indirect sonic vibration to flowable bulk-fill resin-based composite in Class-II cavities or root canal access cavities.
- The results suggest a potential concern regarding high frequency, direct activation of uncured, flowable bulk-fill resin-based composite in Class-II cavities. However, the limitations of this study emphasize the need for larger-scale research to validate the results before clinical recommendations can be made.
- Voids continue to be an issue in flowable, bulk-fill, resin-based composite restorations.

## Data Availability

The datasets used and/or analysed during the current study are available from the corresponding author on request.
